# A_2B _adenosine receptor activity is reduced in neutrophils from patients with systemic sclerosis

**DOI:** 10.1186/ar1468

**Published:** 2004-12-10

**Authors:** Laura Bazzichi, Letizia Trincavelli, Alessandra Rossi, Francesca De Feo, Antonio Lucacchini, Stefano Bombardieri, Claudia Martini

**Affiliations:** 1 Department of Internal Medicine, Division of Rheumatology, University of Pisa, Pisa, Italy; 2 Departments of Psychiatry, Neurobiology, Pharmacology and Biotechnology, University of Pisa, Pisa, Italy

**Keywords:** adenosine, A_2 _adenosine receptors, neutrophils, receptor binding, systemic sclerosis

## Abstract

We conducted the present study to investigate protein expression and functioning of A_2A _and A_2B _adenosine receptors (ARs) in neutrophils of patients affected by systemic sclerosis (SSc). The presence of A_2A _and A_2B _ARs was assessed by immunoblotting using specific antibodies. Equilibrium A_2A _and A_2B _ARs binding parameters were evaluated by radioligand binding assay. Functional studies were conducted to investigate coupling of the A_2B _AR to the adenylyl cyclase pathway. This is the first report of the use of Western blot analysis to confirm the presence of A_2A _and A_2B _ARs in human neutrophils. No significant changes in A_2A _AR binding parameters or expression levels were detected between SSc patients and healthy control individuals. A significant decrease (65%) in the maximum density of A_2B _AR binding sites occurred in SSc neutrophils, whereas no changes in the affinity constant values were found. Moreover, a decrease in A_2B _AR mediated adenylyl cyclase activity was observed in patients with SSc. Our findings demonstrate the occurrence of selective alterations in A_2B _AR density and signalling in SSc.

## Introduction

Systemic sclerosis (SSc), also known as scleroderma, is a connective tissue disease of unknown aetiology. Possibly an autoimmune disorder, it is accompanied in the vast majority of cases by the presence of antinuclear antibodies [1]. SSc may affect virtually any organ of the body, including skin, gastrointestinal tract, lungs, heart, kidneys, and musculoskeletal system. Altered connective tissue metabolism can cause either localized or diffuse thickening of the skin, while inflammation is associated with endothelial damage. Clinically, microvascular disturbance, teleangiectasia, Raynaud's phenomenon, polyarthralgia and polyarthritis, as well as oesophageal hypomobility, visceral muscolaris mucosa damage and pulmonary fibrosis, have been described [2].

The mechanisms leading to endothelial damage, inflammation and fibrosis are unclear. Reactive oxygen species in neutrophils may increase the extent of inflammation and fibrosis during the respiratory burst and could be involved in endothelial damage [3]. The endothelial cells of microvessels are deficient in the synthesis of catalase, which provides natural defence against superoxide damage, and appear to be particularly susceptible to superoxide injury during reperfusion [4].

Adenosine is an important endogenous regulator of neutrophil functioning. It is released intracellularly and modulates neutrophil activity by interacting with specific surface receptors [5]. Distinct adenosine receptor (AR) subtypes A_1_, A_2A_, A_2B _and A_3 _have been identified and their functions characterized in neutrophils. Specifically, activation of A_1 _ARs enhances chemotaxis, phagocytosis and adherence [6,7]; A_2A _ARs inhibit reactive oxygen species generation, phagocytosis and adherence [8-10]; and A_2A _and A_3 _ARs inhibit neutrophil degranulation [11-14]. Adenosine has been shown to prevent the release of vascular endothelial growth factor from neutrophils via A_2B _AR activation [15]. Because activation of ARs reduces both immune and inflammatory responses, adenosine release has been hypothesized to be a possible mechanism of cell self-protection from activated neutrophils [5]. An increase in adenosine deaminase activity has been described in patients with SSc, suggesting an alteration in adenosine control mechanisms in this disease [16,17].

In the present study we analyzed A_2A _and A_2B _AR subtypes in neutrophils from patients affected by SSc by means of expression analysis, radioligand binding assays and functional studies.

## Methods

### Chemicals and reagents

Bacitracine, benzamidine, trypsin inhibitor, sodium orthovanadate, Nonidet P-40, SDS, phenylsulfonylfluoride, aprotinin and adenosine deaminase (ADA) were purchased from Sigma (St. Louis, MO, USA). Unlabelled AR agonists/antagonists and the anti-β-actin antibody were supplied by RBI/Sigma (St. Louis, MO, USA). [^3^H]CGS_21680 _(CGS_21680 _= [2-p-(2-carbowyethyl)phenylethylamino]-5'-N-ethylcarboxamidoadenosine), [^3^H]NECA (NECA = 5'-N-ethylcarboxamidoadenosine), and [^32^P]α-ATP were supplied by NEN Life Sciences (Köln, Germany). Electrophoresis reagents were purchased from BioRad (Munchen, Germany). A_2A_AR and A_2B_AR antibodies were supplied by Alpha Diagnostic (San Antonio, TX, USA). All other chemicals were from standard commercial sources.

### Patients

Twenty-six patients affected by SSc were included in the study (22 women and 4 men; mean age ± standard deviation 53.0 ± 11.3 years). They all fulfilled standard criteria of the American College of Rheumatology for SSc. Sixteen patients were anticentromere antibody positive and four were SCL-70 positive. Limited symptoms of disease, involving skin thickness alterations to the face, hands and feet, were present in 18 patients (mean disease duration <5 years, skin score range [according to the modified Rodnan total skin thickness score] 10–21). Diffuse symptoms with more extensive skin involvement were present in eight patients (mean disease duration <5 years, total skin thickness score range 27–30). The activity score [18] varied between 0.5 and 3.5 and the severity score [19] between 2 and 6. The erythrocyte sedimentation rate was 24 ± 23 mm/hour (mean ± standard deviation).

Control samples were obtained from 26 healthy volunteers, who were similar to the patients included in the study in terms of sex distribution and age (20 women and 6 men; mean age ± standard deviation 49.0 ± 9.2 years). Informed consent to participate in the study was obtained from all individuals.

### Sample collection and neutrophil preparation

Venous blood (20 ml) was drawn between 08:00 and 09:00 a.m. from fasting individuals by antecubital venipuncture, collected in heparinized (10 IU/L) plastic tubes and processed immediately. Neutrophils were isolated following the Boyum method [20] with some modifications.

### Western blot analysis

Neutrophils were lysed in RIPA buffer (150 mmol/l NaCl, 50 mmol/l Tris-HCl, pH 8, 0.5% sodium deoxhycolate, 1% Nonidet P-40, 1 mmol/l phenylsulfonylfluoride, 10 μg/ml aprotinin, 100 μmol/l sodium orthovanadate) for 1 hour at 4°C. After centrifugation at 15,000 *g *for 30 min, soluble fractions were assayed for protein content using BioRad protein assay. Equivalent amounts of proteins (50 μg/sample) were analyzed by SDS-PAGE, using 10% (weight/vol) polyacrylamide resolving gels. Protein bands were transferred to nitrocellulose and probed with 0.1 μg/ml rabbit anti-human A_2A _AR or A_2B _AR antibodies.

A_2A _AR antibody is an affinity-purified rabbit polyclonal antibody raised against a peptide mapping to the carboxyl-terminus of A_2A _AR. It specifically reacts with human, bovine, rat and pig A_2A _receptors and does not cross-react with A_1_, A_2B_, or A_3 _AR subtypes. A_2B _AR antibody is an affinity-purified rabbit polyclonal antibody raised against a region that corresponds to the second extracellular domain of A_2B _AR of human origin.

After washing, membranes were incubated with anti-rabbit secondary antibody conjugated to horseradish peroxidase for 2 hours at room temperature, and bands were visualized by chemiluminescence, in accordance with the manufacturer's instructions (Sigma-Aldrich). Membranes were re-probed with an anti-β-actin antibody for normalization.

### Binding assay

For membrane preparation, cells were washed twice with 10 mmol/l Tris-HCl buffer, pH 7.4, containing 10 mmol/l MgCl_2_, in the presence of protease inhibitors (200 μg/ml bacitracine, 160 μg/ml benzamidine, 20 μg/ml trypsin inhibitor [T1]) and centrifuged at 48,000 *g *for 15 min at 4°C. Pellets were diluted in 20 volumes of T1 buffer, treated with ADA (2 IU/ml) for 60 min at 37°C to remove endogenous adenosine, and washed twice with 50 mmol/l Tris-HCl buffer, pH 7.4, containing 10 mmol/l MgCl_2 _(T2).

A_2A _AR binding assay was performed by using a specific radiolabelled A_2A _AR agonist, namely [^3^H]CGS_21680_. Aliquots of neutrophil membranes (0.2–0.3 mg protein) were incubated with different [^3^H]CGS_21680 _concentrations (5–30 nmol/l) in a final volume of 250 μl of T2 buffer. Nonspecific binding was determined in the presence of 100 μmol/l NECA. After 90 min incubation at 25°C, the binding reaction was terminated by vacuum filtration through Whatman GF/C glass fibre filters (Whatman, Maidstone, UK), accompanied by three washes with ice-cold T2 buffer (4 ml). A_2A _AR specificity was evaluated through competition experiments, using different AR ligands.

A_2B _AR binding assay was performed using 20 nmol/l [^3^H]NECA in the presence of 50 nmol/l cyclopentyladenosine (CPA) and 100 nmol/l SCH_58261 _(SCH_58261 _= 5-amino-7-[phenylethyl]-2-[2-furyl]-pyrazolo [4,3-e]-1,2,4-triazolo [1,5-c]pyrimidine) to prevent [^3^H]NECA binding to A_1 _and A_2A _ARs, respectively [21]. Scatchard analysis was performed on competition experiments carried out in the presence of unlabelled NECA at concentrations ranging from 50 nmol/l to 2 mmol/l. Aliquots of neutrophil membranes (0.2–0.4 mg proteins) were incubated in a final volume of 250 μl T2 buffer. Nonspecific binding was evaluated in the presence of 100 μmol/l NECA. After 90 min incubation at 0°C, the reaction was terminated either by vacuum filtration through Whatman GF/C glass fibre filters, accompanied by three washes with ice-cold T2 buffer (4 ml), or by centrifugation at 2900 *g *for 15 min at 4°C. A_2B _AR specificity was evaluated through competition experiments, using different AR ligands.

### Adenylyl cyclase assay

Neutrophils were homogenized in buffer solution containing 10 mmol/l Hepes, 1 mmol/l EGTA and 10 mmol/l NaCl_2_, and then centrifuged at 46,500 *g *for 20 min at 4°C. Pellets were resuspended in 10 volumes of 10 mmol/l Hepes, containing protease inhibitors (200 μg/ml bacitracine and 160 μg/ml benzamidine), incubated for 30 min at 30°C with 2 U/ml ADA, and centrifuged. Adenylyl cyclase (AC) activity was measured as described by Salomon [22] and Johnson and Salomon [23], with some modifications. NECA-mediated stimulation of AC activity was assessed by incubating aliquots of membranes with increasing NECA concentrations from 0.01 nmol/l to 10 μmol/l. The reaction was started by adding membrane aliquots (10–50 μg proteins/tube), conducted for 15 min at 24°C, and then stopped by transferring samples on ice and adding 500 μl ice-cold stop solution (120 mmol/l zinc acetate, 144 mmol/l Na_2_CO_3_). The stop solution contained [^3^H]cAMP (10,000–15,000 cpm/sample) to monitor column recovery. Newly formed ZnCO_3 _allowed precipitation of residual ATP, discarded through centrifugation at 2700 *g *for 8 min. Supernatants containing both [^32^P]α-cAMP and [^3^H]cAMP were further purified by double-step Dowex-Alumina chromatography and counted by means of a β-counter (Packard Tricarb 1600; Perkin Elmer, Wellesley, MA, USA).

To evaluate A_2B _AR mediated cAMP accumulation, the reaction was carried out in the presence of selective A_2A _antagonist SCH_58261 _at a concentration (100 nmol/l) able to block A_2A _receptors completely [21].

### Data and statistical analysis

Affinity constant values (Kd) and maximum number of binding sites (B_max_) were calculated using the nonlinear multipurpose curve-fitting computer program Graph-Pad Prism The 50% inhibitory concentration values were calculated using the same program and converted to Ki values through the Cheng and Prusoff equation.

A GS-670-BIO-RAD imaging densitometer was used for semiquantitative analysis of immunoblots. Partial F test (*P *< 0.01) was used to determine binding data with the best fit to a one-site or two-site model. Differences in binding parameters between SSc patients and control individuals were evaluated by one-way analysis of variance.

## Results

**Figure 1:**
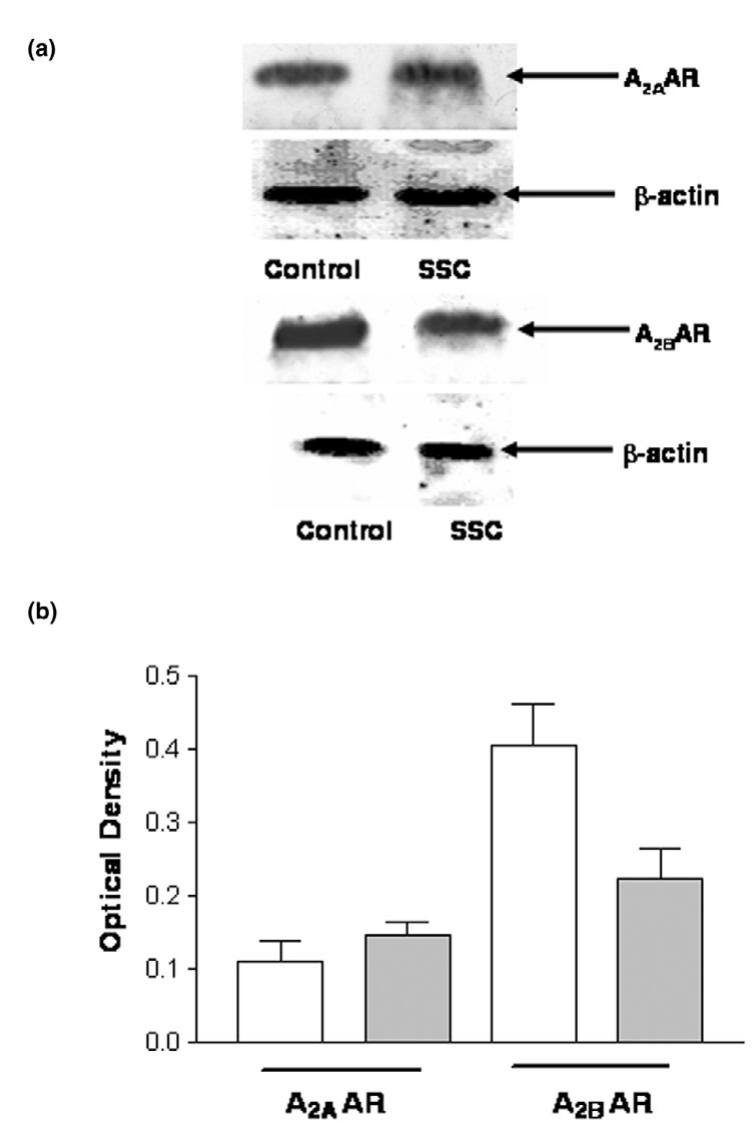
Immunoblotting analysis of A_2A _and A_2B _adenosine receptors (ARs) from systemic sclerosis (SSc) neutrophils and controls. Cells obtained from 26 healthy volunteers and 26 SSc patients were lysed as described in the Methods section. Equal amounts of protein (50 μg) were separated on polyacrylamide gel, blotted and probed with 0.1 μg/ml rabbit anti-human A_2A _AR or A_2B _AR antibodies. Immunoreactive bands were visualized according to electrogenerated chemiluminescence protocol. A_2A _and A_2B _AR antibodies recognized immunoreactive bands of 45 kDa and 50 kDa, respectively. **(a) **Representative experiment performed on neutrophils from one healthy volunteer and one SSc patient. **(b) **Densitometric analysis of A_2A _and A_2B _AR immunoreactive bands from 26 healthy volunteers and 26 SSc patients. Graph bars: mean ± standard error of band density, normalized to β-actin. White bars are controls; grey bars are SSc patients.

In both control and SSc neutrophils, Western blot analysis identified two specific immunoreactive bands of 45 kDa and 50 kDa, corresponding to A_2A _and A_2B _ARs, respectively (Fig. 1). This confirmed the presence of both AR subtypes in human neutrophils.

To characterize ARs, binding assays were conducted in neutrophil membrane fractions. SSc patients were randomly divided into two subgroups in order to obtain large amounts of protein, as required by the experiments.

**Figure 2:**
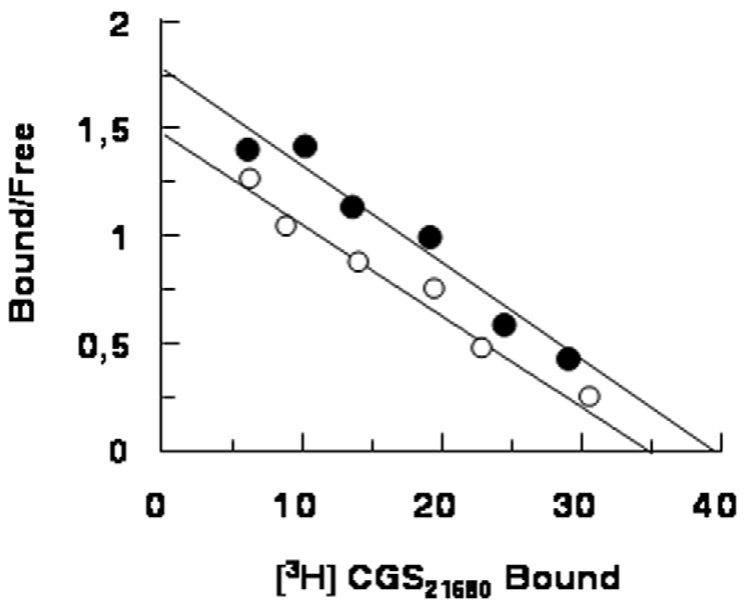
Representative Scatchard plot of [^3^H]CGS_21680 _saturation binding data. Empty circles indicate neutrophil membranes from healthy volunteers (affinity constant [Kd] = 25 ± 1.3 nmol/l; maximum number of binding sites [B_max_] = 35 ± 2.4 fmol/mg); filled circles indicate neutrophil membranes from systemic sclerosis (SSc) patients overall (Kd = 23 ± 1.8 nmol/l; B_max _= 40 ± 3.2 fmol/mg). Assays were performed in triplicate.

The selective A_2A _AR agonist [^3^H]CGS_21680 _identified a homogenous population of binding sites in control individuals. Kd and B_max _values were 25 ± 1.3 nmol/l and 35 ± 2.4 fmol/mg protein, respectively (Fig. 2). Competition experiments using [^3^H]CGS_21680 _in combination with a variety of A_2A _ligands revealed a pharmacological profile typical for A_2A _ARs (R-PIA [R-N6-phenylisopropyladenosine] > teofilline > NECA > SCH_58261_; data not shown). Scatchard analysis for SSc neutrophils revealed no significant differences in Kd and B_max _between patients (mean values: Kd = 23 ± 1.8 nmol/l, B_max _= 40 ± 3.2 fmol/mg protein) and healthy control individuals (*P *> 0.05; Fig. 2), suggesting that no alteration in A_2A _binding sites occurs in SSc. In agreement with this, densitometric analysis of immunoblots showed no significant changes in A_2A _AR immunoreactive bands in SSc neutrophils relative to controls (optical density: 0.11 ± 0.03 for patients versus 0.15 ± 0.02 for controls).

**Figure 3:**
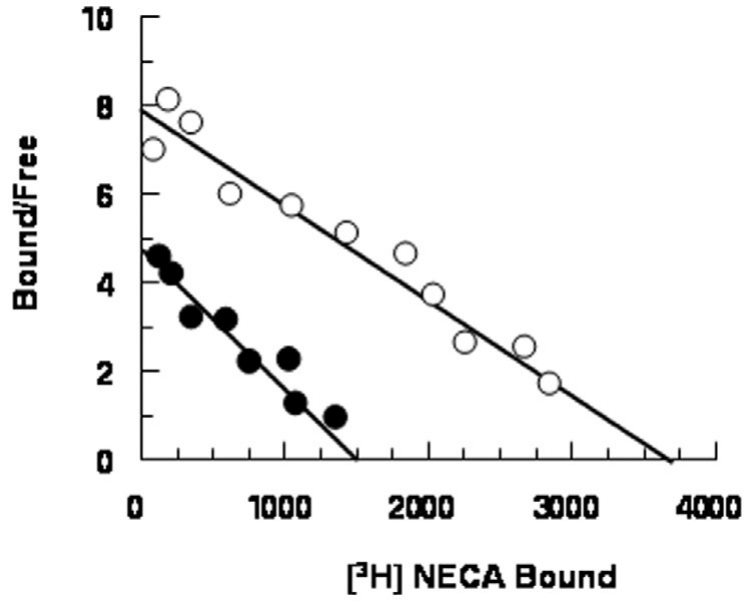
Representative Scatchard plot of [^3^H]NECA saturation binding data. Competition binding experiments were performed, incubating aliquots of neutrophil membranes with 20 nmol/l [^3^H]NECA and different NECA concentrations (50 nmol/l to 2 mmol/l), in the presence of 50 nmol/l CPA and 100 nmol/l SCH_58261_. Empty circles indicate neutrophil membranes from healthy volunteers (affinity constant [Kd] = 476 ± 34 nmol/l, maximum number of binding sites [B_max_] = 3696 ± 210 fmol/mg); filled circles indicate neutrophil membranes from systemic sclerosis (SSc) patients overall (Kd = 469 ± 35 nmol/l, B_max _= 1292 ± 98 fmol/mg). Assays were performed in triplicate.

**Figure 4:**
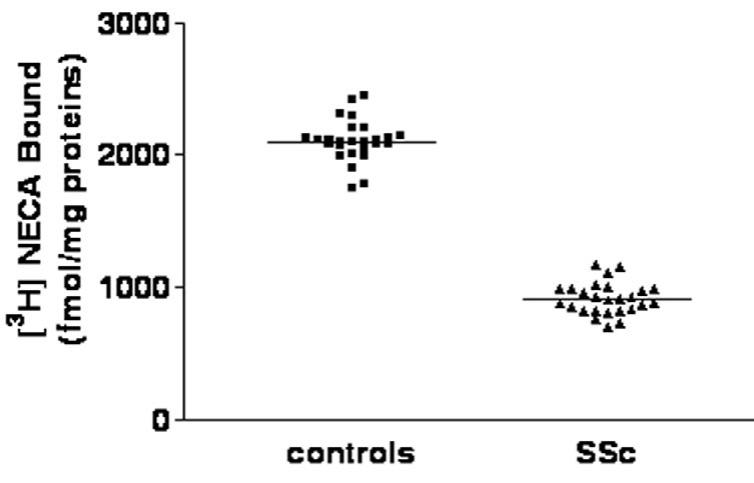
A_2B _adenosine receptor binding experiments performed in individual patients using NECA at 500 nmol/l concentration. Neutrophils were obtained from healthy volunteers (*n *= 26) and systemic sclerosis (SSc) patients (*n *= 26). Horizontal lines indicate the mean values.

A_2B _AR binding sites were identified using [^3^H]NECA as radioligand in the presence of 50 nmol/l CPA and 100 nmol/l SCH_58261_, to prevent nonspecific binding to A_1 _and A_2A _AR subtypes. We performed competition experiments using a wide range (50 nmol/l to 2 mmol/l) of [^3^H]NECA concentrations to allow the identification of A_2B _AR low-affinity binding sites. Data analysis revealed that the one-site model produced a significantly better fit than the two-site model (*P *< 0.05), both in control and SSc neutrophils. In our experimental conditions, control neutrophils exhibited the presence of low-affinity binding sites with Kd and B_max _values of 476 ± 34 nmol/l and 3696 ± 210 fmol/mg, respectively (Fig. 3). Competition experiments using [^3^H]NECA in combination with a variety of AR ligands revealed a pharmacological profile typical for A_2B _ARs (R-PIA > teofilline > SCH_58261 _= MRS1220 > DPCPX > 2Cl-adenosine > NECA > MRS1706; Table 1). Scatchard analysis for SSc neutrophils showed no significant differences in Kd and B_max _between the two subgroups of patients. However, a significant alteration in B_max _was found relative to controls, whereas Kd values remained unaltered. Overall, mean values for Kd and B_max _in SSc were 469 ± 35 nmol/l and 1292 ± 98 fmol/mg protein, respectively (*P *< 0.05; Fig. 3). Moreover, experiments conducted in individual patients using a concentration of NECA of 500 nmol/l showed similar specific binding values (expressed as fmol/mg protein), confirming the homogeneity of A_2B _AR sites between SSc subgroups (Fig. 4). The alteration in A_2B _AR levels in SSc patients was confirmed by immunoblotting assay. Densitometric analysis of immunoreactive bands showed a reduction in A_2B _expression in SSc patients (optical density 0.22 ± 0.04) as compared with controls (optical density 0.40 ± 0.06; *P *< 0.05; Fig. 1).

**Figure 5:**
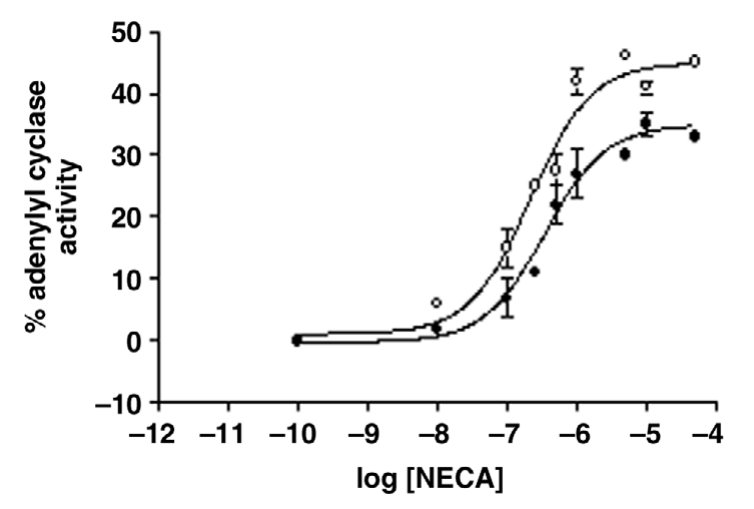
A_2B _adenosine receptor (AR)-mediated stimulation of adenylyl cyclase activity in control (empty circles) and systemic sclerosis (SSc; filled circles) neutrophil membranes. Membranes were incubated with different NECA concentrations (ranging from 10 nmol/l to 100 μmol/l) and the activity of adenylyl cyclase, expressed as pmol/min per mg protein, was evaluated. Values are expressed as mean ± standard error of three indipendent experiments. EC_50 _values were 165 ± 9.3 for control versus 373 ± 26 nmol/l for SSc.

Functional coupling of A_2B _ARs to stimulatory G proteins in neutrophil membranes was assessed by evaluating the effects of the agonist NECA (in the presence of 100 nmol/l SCH_58261_) on AC activity. NECA stimulated AC activity in a concentration dependent manner. Dose-response curves revealed significant differences between SSc patients (EC_50 _= 373 ± 26 nmol/l; E_max _= 35 ± 2.9%) and controls (EC_50 _= 165 ± 9.3 nmol/l; E_max _= 43 ± 3.2%), suggesting an alteration in A_2B _AR responsiveness in SSc (Fig. 5).

## Discussion

In the present study we analyzed A_2A _and A_2B _AR subtypes in neutrophils of patients affected by SSc, by means of Western blot, radioligand binding techniques and functional studies. This is the first report of use of Western blot analysis to confirm the presence of A_2A _and A_2B _ARs in human neutrophils.

A_2A _and A_2B _AR equilibrium binding parameters were measured using radioligand binding assays. Scatchard analysis of [^3^H]CGS_21680 _saturation binding to A_2A _AR showed no significant difference in B_max _or Kd between SSc neutrophils and controls, suggesting that the A_2A _AR subtype remained unaltered in SSc. Conversely, when A_2B _AR was analyzed a reduction in B_max _(65%) was observed, with no significant change in Kd values.

A_2B _ARs are known to be low-affinity adenosine binding sites. Competition experiments using a variety of A_2B _AR agonists and antagonists revealed a pharmacological profile typical of A_2B _ARs, which is consistent with studies conducted in transfected cell models. Our findings represent the first characterization of A_2B _ARs in neutrophils with binding experiments.

In order to analyze a population of nonhomogenous patients and to evaluate the impact of the disease on A_2 _ARs, SSc patients were randomly divided into two subgroups. No difference was found when the two groups were compared, suggesting that different degrees of disease severity and activity had no impact on the assays, but that the disease *per se *is required to modulate levels and functioning of A_2B _receptors.

Functional studies were performed to investigate whether the decrease in level of A_2B _ARs was accompanied by alterations in receptor responsiveness. An evaluation of the ability of NECA to increase AC activity revealed functional coupling of A_2B _receptors to G proteins. In SSc patients a significant reduction (by more than 50%) in NECA potency was observed, without any effect on agonist efficacy.

Our findings suggest that a selective reduction in A_2B _AR levels and responsiveness occurred in SSc. Alterations in the expression and functionality of A_2B _ARs (low-affinity ARs) in patients with SSc may be responsible for the increase in free oxygen radicals, and consequent oxidative damage, that characterizes SSc. This would account for impaired control of hypoxic and inflammatory processes.

In neutrophils it has long been known that adenosine and its analogues inhibit O_2 _^- ^generation, phagocytosis and cell adherence by occupying specific A_2 _ARs. Because hypoxia, ischaemia and inflammation can stimulate adenosine production, A_2 _AR regulation has been postulated to be a self-protective mechanism for cells from activated neutrophils [24]. Eltzschig and coworkers [25] reported that A_2B _ARs are selectively upregulated in endothelial cells by hypoxia (more than fivefold increase in mRNA), which is associated with ATP hydrolysis and release of adenosine. Taken together, these findings show some coordination between AR transcription and nucleoside signalling at the vascular interface during hypoxia. We might speculate that chronic inflammatory conditions in SSc patients impaired regulatory mechanisms mediated by the anti-inflammatory effects of adenosine via A_2B _AR activation. In addition, it was reported by Visser and coworkers [26] that increases in cAMP in activated neutrophils play an anti-inflammatory role. The reduced activation of cAMP we observed in SSc patients might be correlated with the inability of these patients to control the inflammatory process.

It was no surprise to find an alteration in adenosinergic system responsiveness in SSc. In fact, adenosine produces a constellation of responses, including anti-inflammatory actions and vasodilatation, mediated through interactions with high-affinity receptor subtype A_2A _and low-affinity receptor subtype A_2B_. Moreover, in SSc and related disorders, alterations in adenosine metabolism have been suggested. Indeed, purine analogue 2-chlorodeoxyadenosine, which is utilized for the treatment of such chronic disorders [27,28], appears to reduce the number of abnormal fibroblasts.

A_2B _ARs were initially thought to be of lesser physiological relevance because of their relatively low affinity for adenosine, and it was only recently that important functions attributable to A_2B _ARs were discovered. A pivotal role for them was postulated in inflammatory pathological conditions, when adenosine is released at high levels (up to the micromolar range). In light of our findings, a closer examination of A_2B _AR functions may be valuable because of the potential therapeutic importance of these receptors as targets for treatment with selective agents.

## Conclusion

Our findings demonstrated a reduction in A_2 _low-affinity (A_2B_) AR density and functioning in neutrophils of patients affected by SSc, suggesting an alteration in adenosinergic system responsiveness. This reduction could relate to the increased production of free oxygen radicals and consequent oxidative damage that characterize SSc, highlighting an impairment in the ability of neutrophils to control hypoxia and inflammation.

No differences between two randomly selected subgroups of SSc patients were found, thus suggesting that different degrees of disease severity and activity had no impact on the degree of A_2B _AR reduction. Consequently, the functional status of A_2B _ARs may be considered a marker of the disease, making it worthwhile to characterize a larger cohort of patients, including their closest relatives and patients with early SSc.

## Abbreviations

AC = adenylyl cyclase; ADA = adenosine deaminase; AR = adenosine receptor; B_max _= maximum number of binding sites; CGS_21680 _= (2-p-[2-carbowyethyl]pheylethylamino)-5'N-ethylcarboxamidoadenosine; CPA = cyclopentyladenosine; Kd = affinity constant; NECA = 5'-N-ethylcarboxamidoadenosine; R-PIA = R-N6-phenylisopropyladenosine; SCH_58261 _= 5-amino-7-(phenylethyl)-2-(2-furyl)-pyrazolo(4,3-e)-1,2,4-triazolo(1,5-c)pyrimidine; SSc = systemic sclerosis.

## Competing interests

The author(s) declare that they have no competing interests.

## Authors' contributions

LB organized the study design and recruited the patients. LT carried out the binding experiments and statistical analysis. AR participated in the immunoblotting experiments and helped to draft the manuscript. FdF participated in the collection of human samples. AL participated in the coordination of the study and helped with problem solving. SB participated in the coordination of the study and in planning the manuscript. CM participated in the coordination of the study and designed the AC assay. All authors read and approved the final manuscript.

## Figures and Tables

**Table 1:** Specificity of [^3^H]NECA binding to A_2B _adenosine receptors in control neutrophil membranes

	[^3^H]NECA Ki (μmol/l)
NECA	0.315 ± 0.028
2 Cl-adenosine	0.954 ± 0.600
R-PIA	1000 ± 86
SCH_58261_	>10
Teofilline	47 ± 3.5
MRS1706	0.005 ± 0.0003
DPCPX	2 ± 0.12
MRS1220	>10

Competition experiments were performed, incubating aliquots of neutrophil membranes with 20 nmol/l [^3^H]NECA (plus 50 nmol/l CPA and 100 nmol/l SCH_58261_) in the presence of increasing ligand concentrations. Ki values are expressed as mean ± SEM of three separate experiments. Ki values were calculated from IC_50 _values (concentration of drug causing 50% inhibition of specific binding) using the Cheng and Prusoff equation.
